# Bidirectional Differentiation of Human-Derived Stem Cells Induced by Biomimetic Calcium Silicate-Reinforced Gelatin Methacrylate Bioink for Odontogenic Regeneration

**DOI:** 10.3390/biomedicines9080929

**Published:** 2021-07-31

**Authors:** Yi-Ting Lin, Tuan-Ti Hsu, Yu-Wei Liu, Chia-Tze Kao, Tsui-Hsien Huang

**Affiliations:** 1 School of Dentistry, Chung Shan Medical University, Taichung 40201, Taiwan; ytdoctor@gmail.com (Y.-T.L.); ctk@csmu.edu.tw (C.-T.K.); 2 x-Dimension Center for Medical Research and Translation, China Medical University Hospital, Taichung 404332, Taiwan; nakohsu@gmail.com (T.-T.H.); a0934077552@gmail.com (Y.-W.L.); 3 Department of Stomatology, Chung Shan Medical University Hospital, Taichung 40201, Taiwan

**Keywords:** calcium silicate, gelatin methacryloyl, odontogenesis, bioprinting, bioink

## Abstract

Tooth loss or damage is a common problem affecting millions of people worldwide, and it results in significant impacts on one’s quality of life. Dental regeneration with the support of stem cell-containing scaffolds has emerged as an alternative treatment strategy for such cases. With this concept in mind, we developed various concentrations of calcium silicate (CS) in a gelatin methacryloyl (GelMa) matrix and fabricated human dental pulp stem cells (hDPSCs)-laden scaffolds via the use of a bioprinting technology in order to determine their feasibility in promoting odontogenesis. The X-ray diffraction and Fourier transform-infrared spectroscopy showed that the incorporation of CS increased the number of covalent bonds in the GelMa hydrogels. In addition, rheological analyses were conducted for the different concentrations of hydrogels to evaluate their sol–gel transition temperature. It was shown that incorporation of CS improved the printability and printing quality of the scaffolds. The printed CS-containing scaffolds were able to release silicate (Si) ions, which subsequently significantly enhanced the activation of signaling-related markers such as ERK and significantly improved the expression of odontogenic-related markers such as alkaline phosphatase (ALP), dentin matrix protein-1 (DMP-1), and osteocalcin (OC). The calcium deposition assays were also significantly enhanced in the CS-containing scaffold. Our results demonstrated that CS/GelMa scaffolds were not only enhanced in terms of their physicochemical behaviors but the odontogenesis of the hDPSCs was also promoted as compared to GelMa scaffolds. These results demonstrated that CS/GelMa scaffolds can serve as cell-laden materials for future clinical applications and use in dentin regeneration.

## 1. Introduction

Tooth loss or damage is usually due to chronic periodontal diseases, infections, dental caries, traumatic injuries, or even osteoporosis [1]. Excessive tooth loss will cause facial collapse and malocclusion. Therefore, tooth restoration is a huge clinical problem for scientists and physicians alike. Traditionally, tooth restoration includes replacement with artificial materials such as removable partial dentures, fixed partial dentures, or even dental implants. However, artificial dentures often have a high failure rate due to the progression of chronic dental diseases, thus prompting the need for novel tooth restoration methods. Stem cell-based tissue engineering has evolved during the past decade, and recent publications have revealed the successful reconstruction of biological tissues, including bones, cartilage, heart, and blood vessels [2,3,4]. In the field of regenerative dentistry, researchers have explored new methodologies for the treatment of injured dental structures through the development of novel dentin-pulp tissue based on a combination of human dental pulp stem cells (hDPSCs) of oral origin, growth factors, and scaffolds [5]. Among these sources, hDPSCs have been determined to be the best candidate for odontogenesis since they have been reported to have excellent clonogenic capabilities and rapid proliferation rates [6]. In addition, Gronthos et al. demonstrated that not only are hDPSCs multipotent, they have the ability to differentiate into dentinlike odontoblastic tissues when transplanted into immunocompromised mice [7]. Recently, multiple studies have shown that hDPSCs seeded on scaffolds are a promising solution for clinical tooth regeneration [8,9,10].

The identification and development of suitable bioscaffolds capable of supporting hDPSCs and enhancing odontogenesis are critical aspects of tooth engineering [11]. An ideal scaffold should be non-toxic, yet bioactive, and most importantly, it should be able to provide a 3D microenvironment similar to native tissues that supports cell growth, proliferation, and differentiation [12,13,14]. In recent years, several studies demonstrated that the microenvironment of a 3D hydrogel is closer to the real situation than the 2D culture method because it can improve the proliferation and differentiation functions during the cell culture process [15]. Prescott et al. fabricated a hDPSCs-encapsulated collagen I hydrogel with a growth factor dentin matrix protein-1 (DMP-1) that induced formation of dental pulplike tissues from hDPSCs [16]. In addition, gelatin is derived from collagen and is a common biomaterial used in the fabrication of hydrogels [17]. In recent years, several studies modified gelatin with methacryloyl groups (GelMa) to incorporate photocrosslinking so as to allow for customizable mechanical properties [18,19,20]. Smith et al. fabricated 3–5% GelMa hydrogel encapsulated with harvested pig dentin epithelial or hMSCs with human umbilical vein endothelial cells that revealed the GelMa constructs were able to support in vivo dental cell differentiation and vascularization [21].

Calcium silicate (CS) is another group of bioceramics commonly used in clinical pulp regeneration [22]. CS products can be widely found on the market, and their biocompatibility and bioactivity have been widely discussed in the literature in the past decade [9,23,24]. CS had been shown to release Si ions, which have been proven to enhance vascularization by inducing the secretion of angiogenic-related factors from nearby fibroblasts [25]. Furthermore, the release of Si ions has also been found to promote hard tissue regeneration and to enhance odontogenic differentiation in various types of stem cells, notably hDPSCs [9]. Recently, several studies combined CS with other natural hydrogels in order to better mimic the architecture of native dentin. Qu et al. fabricated a gelatin with bioglass hybrid scaffold and showed that such a modification provided an improved microenvironment for odontogenic differentiation of hDPSCs [26]. The release of calcium ions around CS scaffolds were reported to contribute to the adhesion of tissue–scaffold interfaces via chelation reactions, thus leading to enhanced tissue regeneration [27]. Previously, we successfully fabricated Si-infused GelMa hydrogels. The results showed that these Si-infused GelMa hydrogels were superior to pure GelMa hydrogels in both in vitro and in vivo studies [15]. However, to the best of our knowledge, there are currently no studies reporting on GelMa in combination with CS powder for use in odontogenesis.

In this study, we developed various concentrations of CS into GelMa scaffolds that were cross-linked using UV light. Taken together, it was concluded that CS can be successfully added into GelMa and that such a modification further increases printability, mechanical properties, Si ion secretion, and in vitro odontogenesis. Therefore, we believed that the development of CS/GelMa-based bioink for tissue engineering is still incomplete and that further novel modifications and improvements are still required to make GelMa a suitable biomatrix for dental regeneration engineering. The findings of this study, however, suggest that the use of CS/GelMa could be a potential method that could be used in future dental tissue engineering and clinical applications.

## 2. Materials and Methods

### 2.1. Preparation of the CS/GelMa Bioink

Type A gelatin powder (Sigma-Aldrich, St. Louis, MO, USA) was dissolved in 50 °C phosphate buffer saline (PBS, Invitrogen, Carlsbad, CA, USA) to obtain a 10% gelatin solution. The methacrylic anhydride (Ma, Sigma-Aldrich, St. Louis, MO, USA) was dripped into the gelatin solution (Ma/gelatin ratio = 0.6) and left to react for 3 h, after which the GelMa solution was centrifuged at 40 °C at 8000 rpm and further dialyzed for 24 h to remove any unreacted Ma. The remaining GelMa solution was then lyophilized for 72 h and stored at −20 °C for further usage. Then, GelMa was first dissolved in deionized water to obtain a 15% GelMa solution. We added 0.25% of the LAP photoinitiator (Sigma-Aldrich, St. Louis, MO, USA) to the GelMa solution and left to react in the dark after which 0%, 5%, or 10% (*v*/*w*) calcium silicate powder (CS, Sigma-Aldrich, St. Louis, MO, USA) was added into the GelMa solution and stirred for 1 h.

### 2.2. Shear Rheology Analysis

The rheological properties of the CS/GelMa bioink solution were analyzed using a rheometer (MCR 302, Anton Paar, Graz, Austria). For the shear rheology analysis, the bioink was first dripped onto 50 mm plate heated to 37 °C and exposed to a 1 Hz oscillation frequency, after which the temperature was decreased from 37 ℃ to 15 ℃ within 20 min to determine the sol–gel temperature of the bioink. In a separate study, the bioink on the 50 mm plate heated to 37 °C was exposed to 365 nm UV irradiation (10 mW/cm^2^) for 180 s to allow UV photocuring. The test was conducted for every 1 °C change in the temperature from 37 ℃ to 15 ℃. The strain curves of the storage modulus (G′) and loss modulus (G″) of each test sample were then recorded.

### 2.3. Bioink Analysis

An X-ray diffraction analyzer (XRD; Bruker D8 SSS, Karlsruhe, Germany) was used to evaluate the composition of the CS/GelMa bioink. In addition, Fourier-transform infrared spectroscopy (FTIR, Bruker, Germany) was used to evaluate the presence of functional groups in the range of 4000–400 cm^−1^. An EZ-Test kit (Shimadzu, Kyoto, Japan) was used to obtain the stress–strain profile of the scaffold according to the ASTM D638 guidelines. The guideline stipulates the shape, size, and thickness of the scaffolds to be tested. The specimens were designed in the shape of a dumb-bell with the two ends clamped to the machine. A tensile test speed of 1 mm/s was applied from both ends until the scaffold was torn from the middle. Six scaffolds from each group were tested, and the experimental results were plotted with distance (mm) and load (N).

### 2.4. Printability of Scaffold Fabrication

The scaffolds used in this study were fabricated using an extrusion 3D printer (BioX, Cellink, Gothenburg, Sweden) with a 30 G nozzle set at a printing pressure of ~180 kPa and at a printing rate of 20 mm/s. The printed scaffold was then cured using 405 nm UV light for 90 s.

### 2.5. In Vitro Immersion Test

For this test, the CS/GelMa scaffolds were immersed in simulated body fluid (SBF) to evaluate the weight loss and ion release profiles of the scaffolds. The composition of SBF used in this study is similar to that of human plasma, and its formulated compounds include 7.9949 g NaCl, 0.2235 g KCl, 0.147 g K2HPO4, 0.3528 g NaHCO3, 0.071 g Na2SO4, 0.2775 g CaCl2, and 0.305 g MgCl2·6H2O. These compounds were dissolved in 1000 mL of distilled water in order, and Tris buffer and HCl were added at the end to adjust the pH to 7.4. The scaffolds from each group were soaked in 37 °C SBF for a fixed period of time, and the degradation rate was calculated using the following formula:

Degradation rate (%) = (W0 − Wt)/W0 × 100%.

In addition, inductively coupled plasma atomic emission spectroscopy (ICP-AES, Perkin-Elmer OPT 1MA 3000DV, Shelton, CT, USA) was used to measure the amount of Si ion released after periods of immersion.

### 2.6. Cell Viability and Morphology

The human dental pulp stem cells (hDPSCs) used in this study were purchased from Lonza (PT-5025, Lonza, Basel, Switzerland) and cultured with a commercially available human dental pulp stem cell bullet kit (PT-3005, Lonza, Basel, Switzerland) MSCM commercial medium to passage 4–7 in a 37 °C humidified atmosphere with 5% CO_2_. For the cellular-related studies, 5 × 10^6^ cells/mL were encapsulated into the GelMa solution prior to printing. The fabricated cell-laden scaffold was cultured in medium and replaced every 3 days. At 1, 3, and 7 days of culture, PrestoBlue^®^ (Invitrogen, Carlsbad, CA, USA) was used to detect the cell viability. Briefly, the scaffolds were rinsed with PBS, and PrestoBlue was added and reacted in a 37 °C incubator for a period of 3 h. Then, 100 μL of the mixture solution was transferred to a 96-well microplate. A spectrophotometer (Infinite Pro M200, Tecan, Männedorf, Switzerland) was used to read the absorbance at a wavelength of 570 nm with a reference wavelength of 600 nm. The procedures were performed in triplicate.

After 1 and 7 days of culture, fluorescence staining was performed to examine the cell morphology. Firstly, the cultured medium was removed, and gently rinsed with PBS to remove excess medium. The constructs were then fixed with 4% paraformaldehyde for 30 min and permeated with 0.1% Triton X-100 for 30 min. Then, the phalloidin (Alexa Fluor 488, Invitrogen, Carlsbad, CA, USA) ratio 1:500 was added for 2 h in the dark to investigate cytoskeleton (F-actin), the scaffolds were gently rinsed with PBS solution to remove excess solution, and DAPI fluorescent dye was added and left to react for 20 min in the dark. A confocal microscope (Leica TCS SP8, Wetzlar, Germany) was utilized to observe the cells in the dark state.

### 2.7. Western Blot

After being cultured for 1 day, hDPSCs were lysed using Nonidet-P40 buffer (NP40, Sigma-Aldrich, St. Louis, MO, USA), and a BCA protein assay (Thermo Fisher Scientific, Waltham, MA, USA) kit was used to assess for the protein concentrations of different CS/GelMa bioink. Sodium dodecyl sulfate (SDS)-polyacrylamide gel electrophoresis was used to segregate the cell lysates (40 μg protein), which were then transferred onto polyvinylidene difluoride (PVDF) membranes (Merck Millipore, Burlington, MA, USA). These were placed in a solution containing 2% bovine serum albumin and Tris-buffered saline with Tween-20 for 1 h before they were exposed to primary anti-phospho extracellular signal-regulated kinase 1/2 (pERK, 1:1000), anti-extracellular signal-regulated kinase 1/2 (ERK, 1:1000), anti-p38 (1:1000), anti-phospho p38 (pp38, 1:1000), and β-actin (1:5000, Abcam, Cambridge, MA, USA) for 2 h for further immunoblotting. Thereafter, horseradish peroxidase (HRP)-conjugated secondary antibodies were incubated with the samples for 1 h to enable chemiluminescence visualization. For this study, protein expression levels were normalized to β-actin by Image J, the free software of bioanalysis. All experiments were performed in triplicate.

### 2.8. Odontogenesis Differentiation Assay

In order to evaluate for osteogenic capabilities, the scaffolds were cultured in an osteogenic differentiation culture medium (StemProTM osteogenesis differentiation kit, Invitrogen, Carlsbad, CA, USA) for 3, 7, and 14 days. Then, NP40 Cell Lysis Solution (Sigma-Aldrich, St. Louis, MO, USA) was added to the scaffolds and centrifuged at 6000 rpm for 15 min for cell lysis, after which pNPP (Sigma-Aldrich, St. Louis, MO, USA) was added, and the absorbance was assessed using a 405 nm wavelength spectrophotometer to measure the alkaline phosphatase (ALP) level. The measured absorbance was standardized with the protein quantitative detection reagents (BCA, Thermo Fisher Scientific, Waltham, MA, USA). In addition, the production of dentin matrix protein-1 (DMP-1, MBS167298, MyBioSource, San Diego, CA, USA) and osteocalcin (OC, ab195214, Abcam, Cambridge, MA, USA) proteins secretion from the hDPSCs were determined using these ELISA kits, following the manufacturer’s instructions. The protein concentrations were measured based on correlations with a standard curve. All experiments were performed in triplicate. To further clarify ERK 1/2 and p38 inhibitor effect on cell differentiation, the culture medium was added with PD98059 (ERK 1/2 inhibitor, Invitrogen, Carlsbad, CA, USA) and SB203580 (p38 inhibitor, Invitrogen, Carlsbad, CA, USA). After culture, cell differentiation behaviors were evaluated by examining ALP, DMP-1, and OC, as described previously.

### 2.9. Mineralization

The formation of mineralized nodules and calcium deposition on the surface of the scaffold were measured after 7 and 14 days of culture. Firstly, the cells were fixed with 4% paraformaldehyde (Sigma-Aldrich, St. Louis, MO, USA) for 15 min and then stained with 0.5% Alizarin Red S (Sigma-Aldrich, St. Louis, MO, USA) for 30 min at pH 4.2. A confocal microscope was utilized to observe the mineralization cells in the dark state.

### 2.10. Statistical Analyses

A one-way variance statistical analysis and Scheffe’s multiple comparison test were used in this study to evaluate for differences between each group and scaffold. A value of <0.05 was considered to be statistically significant.

## 3. Results and Discussion

### 3.1. Synthesis and Characterization of the CS/GelMa Scaffold

**Figure 1:**
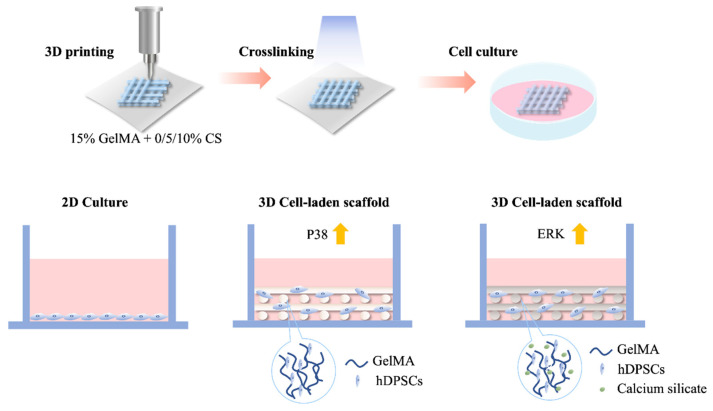
Schematic diagram depicting the fabrication of CS/GelMa scaffolds and advantages of hDPSCs-laden CS/GelMa scaffolds for odontogenesis.

A schematic diagram depicting the fabrication of the CS/GelMa scaffolds is shown in Figure 1. The GelMa was prepared according to established protocols, as reported previously [19], after which photoinitiator LAP and various concentrations of CS powder were added into the GelMa solution to fabricate a photopolymerizable CS/GelMa scaffold. The CS/GelMa were then loaded into an extrusion 3D printer and extruded via a 30 G nozzle with a printing pressure of ~180 kPa and a printing rate of 20 mm/s. The printed scaffolds were then cured using 365 nm UV light for 90 s. GelMa is a common biomaterial used in tissue engineering due to its excellent biological characteristics and tunable physical properties. In addition, gelatin is derived from native collagen and thus closely resembles the native extracellular matrix because it has cell-attaching and matrix metalloproteinase-responsive motifs. Gelatin coupled with methacryloyl further has the advantage of making the physical properties of the hydrogel tunable by adjusting the concentrations of methacryloyl and the irradiation criteria. Another important characteristic of a GelMa hydrogel is that different cell types can be encapsulated into the hydrogel, and the degradation rate can be controlled to match the tissue regeneration rate. However, the main drawback is that GelMa often have low mechanical properties which makes them less suitable for hard tissue engineering than other alternatives.

**Figure 2:**
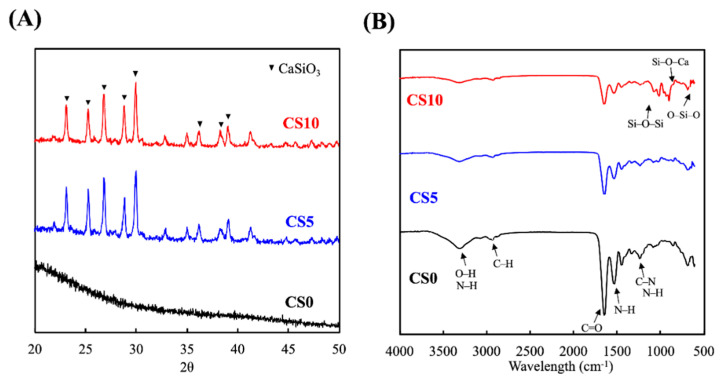
(**A**) X-ray diffractometry (XRD) and (**B**) Fourier-transform infrared spectrometer (FTIR) spectra of the CS/GelMa scaffolds.

The XRD and FTIR analyses of the CS0, CS5, and CS10 scaffolds are shown in Figure 2. Obvious CaSiO_3_ diffraction peaks were observed at 24.5 °C, 25.0 °C, 26.5 °C, 29.4 °C, and 30.5 °C in both CS5 and CS10. On the other hand, there were no such peaks observed in the CS0 group (Figure 2A). Similar to results published by others, the presence of these peaks corresponded to CS and thus indicated that CS was successfully incorporated into the GelMa [28]. FTIR was performed to further confirm the presence of CS in the GelMa and to characterize the interactions between the CS and GelMa. As shown in Figure 2B, CS10 had Si-O-Si, Si-O-Ca, and O-Si-O peaks at 1150, 1000, and 650 cm^−1^, respectively, whilst CS0 had C-O, N-H, and C-N peaks at 1650, 1550, and 1250 cm^−1^, respectively [29]. These results indicated that CS was successfully incorporated into the GelMa via a covalent bond and that different concentrations of CS were also successfully loaded, as seen from the difference in intensity in both CS5 and CS10. In addition, CS10 retained the original structural characteristics of GelMa, which is an important factor to note since the goal was to retain the original superior characteristics of GelMa and yet add the benefits of CS. Furthermore, the presence of additional covalent bonds from the CS loading could potentially enhance the mechanical strength of the hydrogel, thus making it more suitable for hard tissue regeneration.

**Figure 3:**
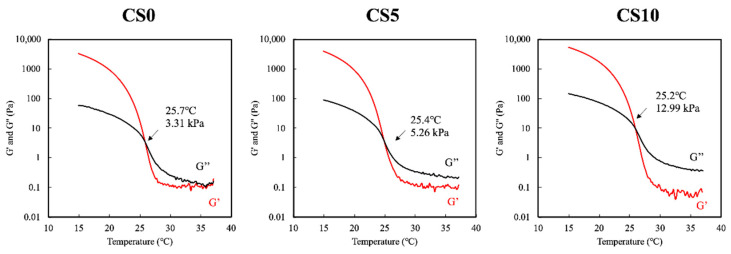
Rheological analyses of CS0, CS5 and CS10. The intersection between storage modulus G′ and loss modulus G″ was regarded as the sol–gel transition temperature.

A rheometer with a cone-plate probe was used to evaluate the sol–gel transition temperature of the CS/GelMa bioink. The various biomaterials were cooled from 40 °C to 10 °C, and the storage modulus (G′) and loss modulus (G″) were recorded as the measurements of the sol–gel characteristics. Simultaneously, a 1 Hz vibration and a strain of 0.25% were administered to the various biomaterials. The intersection between G′ and G″ was determined to be the sol–gel transition temperature. The results of the rheological analyses are shown in Figure 3. Increasing concentrations of CS led to a decrease in the transition temperature with CS0, CS5, and CS10 having temperatures of 25.7 °C, 25.4 °C, and 25.2 °C, respectively. The determination of the sol–gel transition temperature is important since the quality of the bioprinting of hydrogels depends on the transition temperature [30]. A good hydrogel or scaffold should be firm and should be able to be formed into different shapes according to the needs of the researcher. A printing temperature higher than the sol–gel transition temperature will cause the GelMa to turn into a liquid, and vice versa [31].

**Figure 4:**
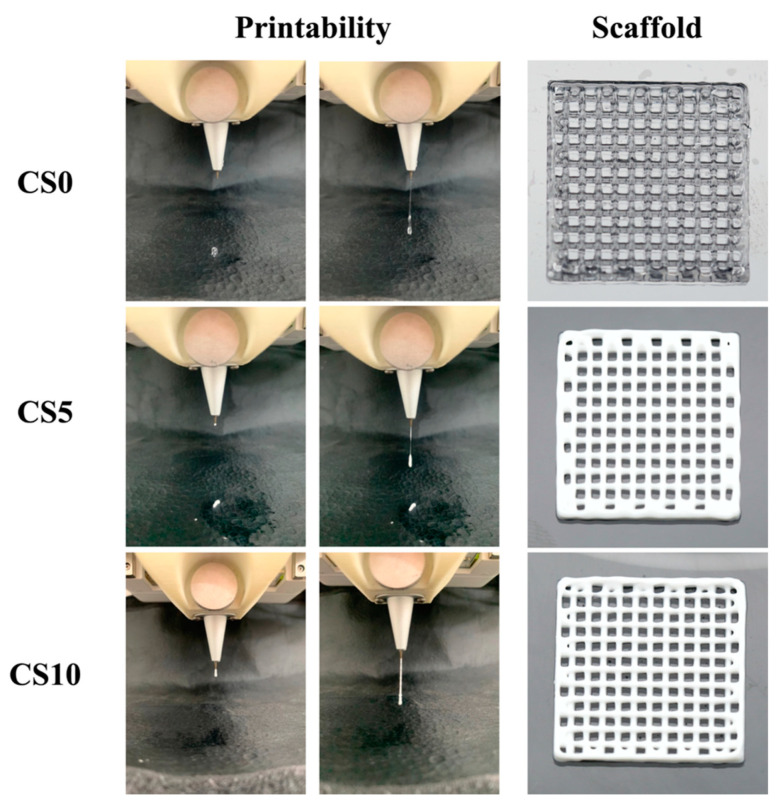
Printability test of CS/GelMa bioinks and the photo image of the fabricated 3D scaffold.

Printability is an important concept in 3D printing since the bioprinted scaffold should attempt to mimic both the cellular architecture and its shape. The printability of the various biomaterials is shown in Figure 4. As shown in Figure 4, the sol–gel transition temperature was adjusted so that all biomaterials could be successfully extruded from the nozzle. In addition, upon application of printing pressure, the biomaterials were again extruded from the nozzles to form a line. As can be seen in the figure, interconnected pores can be seen in all of the scaffolds, and the presence of CS gave the hydrogels a creamy-white appearance. Upon closer observation, it was noted that addition of CS improved the resolution of the scaffolds. The struts and the pores were more well-defined in the CS10 group as compared to the CS0 group. The ability of the bioink to extrude as a consistent filament, the structure of the first layer, rheology behavior, and cross-linking capabilities are all properties that significantly influence bioink printability [32]. In addition, the printability of CS/GelMa bioinks through straight needles was impressed by the adding of CS powder in this study, as shown by the 3D-printed 6-layer scaffold. It is important to note that pore sizes and the interconnectivity of pores play a huge role in guiding and enhancing tissue regeneration [33]. Pore sizes of 200–500 μm have been reported to be optimal for hard tissue engineering [34]. In addition, the interconnectivity of the pores facilitates cellular distribution and integration with host tissues as well as capillary ingrowth. An extrusion-based printing technique was used in this study because extrusion-based printing made it possible to fabricate intricate structures and pores among the scaffolds. During printing, the biomaterial was dispensed by a deposition system controlled by a computer, thus making it possible to control the geometries and obtain precise deposition of the biomaterials. However, one disadvantage of extrusion-based printing is that the biomaterial must have low viscosity to avoid clogging of the needle and yet be controlled at the proper sol–gel temperature range for the proper extrusion of the biomaterials [35]. Given the need for fitting bioink printability and mechanical behaviors of the final scaffolds, CS containing GelMa bioink have been proven to have good potential for use in bioprinting towards possible tissue regeneration applications [36].

**Figure 5:**
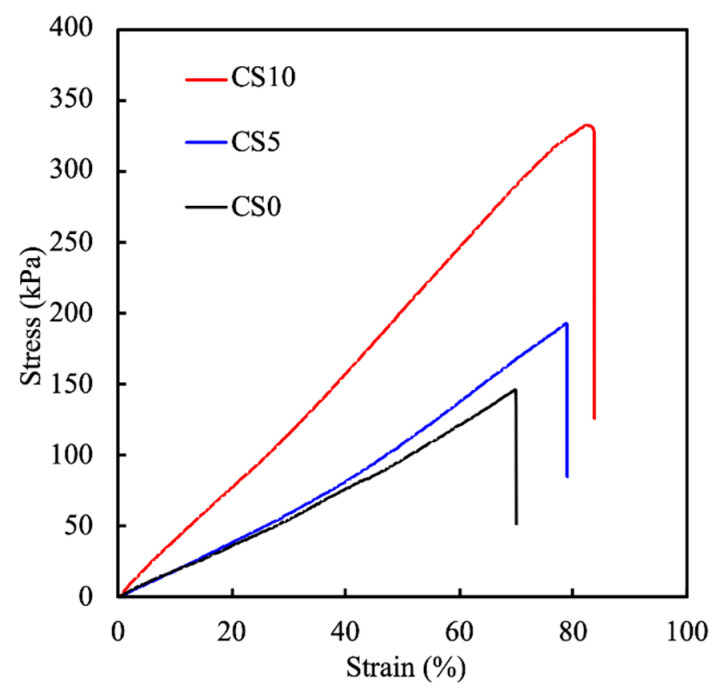
Stress–strain curves of the CS-contained GelMa scaffolds. Data presented as mean ± SEM, *n* = 6 for each group.

The mechanical properties of bioprinted scaffolds are very important for supporting structural integrity and improving cell activity and function [37]. The tensile stress–strain curves and Young’s moduli of the C0, C5, and C10 scaffolds are shown in Figure 5. For this test, the specimens were printed into the shape of a dumb-bell and cured using UV and elastic forces applied on both ends until the scaffolds tore. The stress–strain curves were then plotted with the calculated Young’s modulus. As can be seen, there was a positive correlation between presence of CS, the concentrations of CS, and the mechanical properties of the scaffolds. C0 had a stress–strain and Young’s modulus of 146.1 ± 6.8 kPa, 184.9 ± 6.1 kPa, while C5 and C10 had 193.3 ± 5.7 kPa, 234.4 ± 8.2 kPa and 332.6 ± 10.4 kPa and 413.3 ± 12.5 kPa, respectively. The addition of 5% and 10% CS gave the GelMa a 50% and 150% increase in mechanical strength, respectively. In addition, CS10 had a steeper stress–strain curve as compared to the other groups, thus further indicating that it could withstand a higher strain as compared to the other samples. According to the rheological and mechanical tests, we indicated that the addition of CS powder could significantly enhance the printability compared to GelMa bioink alone, and with the increased concentration of CS powder, the tensile strength increased [38]. It was hypothesized that the increase in stress–strain was due to the increased covalent bonds provided by CS, as shown in the results above. The mechanical properties of scaffolds are known to affect cellular functions, extracellular matrix remodeling, and cellular differentiation [39]. In addition, the increase in tensile stress–strain allows for better manipulation during surgery and for better adaptation to native pressures.

### 3.2. Effects of Degradation Properties on the Soaking Experiments

**Figure 6:**
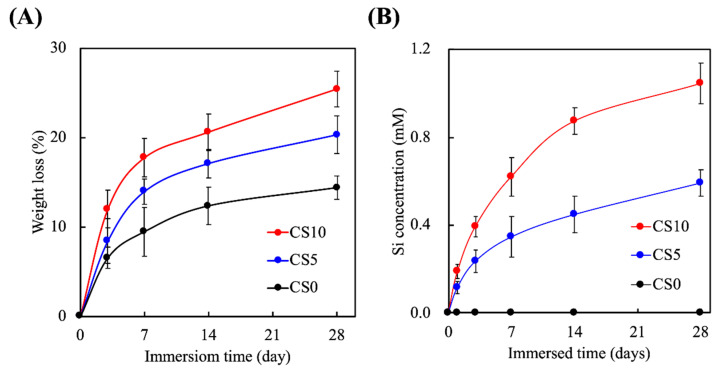
(**A**) Degradation rates and (**B**) release of Si ions of CS0, CS5 and CS10. Data presented as mean ± SEM, *n* = 6 for each group.

The degradation rates of the scaffolds were determined based on the percentage of weight loss after immersion, as shown in Figure 6A. As can be seen, the degradation rates were directly proportional to the percentage of CS present in the scaffolds. All three groups were still intact after 28 days of immersion, with CS0, CS5, and CS10 having a weight loss of 12%, 18%, and 24% after 28 days of immersion, respectively. Furthermore, it could be observed that the majority of the weight loss occurred during the first 7 days of immersion, whereas the degradation rate stabilized from day 7 onwards. The degradation rates of the scaffolds should match the regeneration rates of the host tissues to achieve proper regeneration [40]. In addition, the mechanical integrity of the scaffolds during degradation should be sustainable to allow cellular adhesion and proliferation [41]. In addition, various cellular behaviors require nutrients and growth factors to engage in cell-to-cell interaction in the natural extracellularlike environment provided by the scaffolds. Therefore, even though CS10 had a higher weight loss percentage at 28 days as compared to the rest, it is still worth noting that 75% of the CS10 was still present after 28 days, thus allowing for proper hard tissue regeneration.

The release of Si ions from the scaffolds was evaluated, as shown in Figure 6B. Si ions were only present in CS-contained groups; therefore, the levels of Si ions for CS0 were 0 mM at all timepoints, which was consistent with our hypothesis. On the other hand, increasing the concentrations of CS led to an increased release of Si ions during immersion. CS5 and accumulated 5 mM of Si ions, and CS10 accumulated 10 mM. Similar to the degradation profiles, both CS5 and CS10 had a burst-type release of Si ions into the surrounding fluids for the first 7 days, after which the release of Si ions gradually stabilized. After 28 days of immersion, the scaffolds were still releasing Si ions into the surrounding fluid, which again is an important factor to note since the release of Si ions should be consistent with the rate of bone regeneration [42]. Si ions comprise an important trace element in the human body and are involved in the process of bone regeneration. Recent reports have indicated that Si ions are involved in enhancing proliferation and in the differentiation of stem cells, as well as downstream collagen secretion [43]. Most importantly, it was noted in the present study that the presence of Si ions alone stimulated osteogenic differentiation of stem cells in the absence of osteogenic-inducing factors [44]. Taken together, these findings demonstrated that the addition of CS into GelMa enhanced the mechanical properties and Si ion release capabilities, thus suggesting that these types of scaffolds have great potential for bone regeneration.

### 3.3. In Vitro hDPSCs Culture

**Figure 7:**
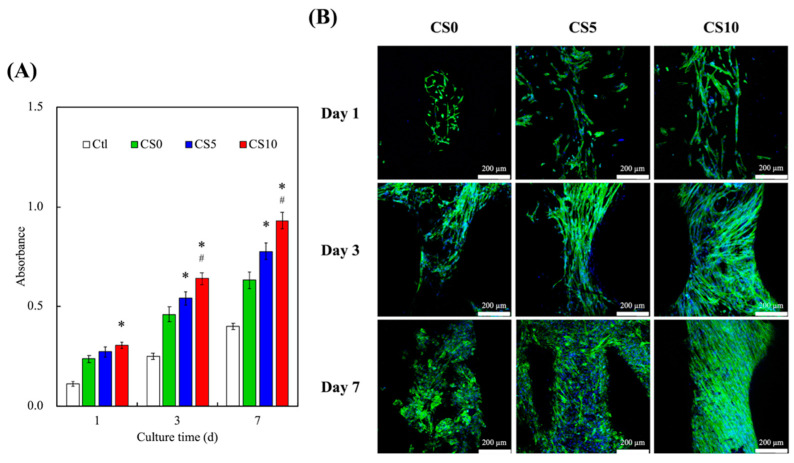
(**A**) Cell proliferation and (**B**) F-actin (green) and DAPI (blue) staining of hDPSCs on the CS-contained GelMa 3D scaffolds at various time points. * indicates a significant difference (*p* < 0.05) when compared to CS0. ^#^ indicates a significant difference (*p* < 0.05) when compared to CS5. The scale bar is 200 µm.

The proliferation rates of the hDPSCs were evaluated over 7 days, and the results are shown in Figure 7A. As can be seen, CS10 exhibited significantly higher cellular proliferation after 1 day of culture as compared to CS0. There were no significant differences in cellular proliferation between CS5 and CS0 on day 1. After 3 days of culture, it was observed that CS10 had significantly higher cellular proliferation as compared to CS5 and CS0. On day 7, CS10 had approximately 50% and 20% higher cellular proliferation as compared to CS0 and CS5, respectively. These results clearly indicated that the CS hydrogel was able to enhance proliferation rates and viability and that increased concentrations of CS resulted in additional increasing effects on both proliferation and viability. In addition, immunofluorescence staining of the cells was carried out, and the results are shown in Figure 7B. As can be seen, there were obviously more cell nuclei in CS10 as compared to all other groups at all time points. Furthermore, on day 1 of culture, the cells on CS10 were flatter and were more evenly-spread as compared to CS0, thus indicating that the microenvironment of CS10 was favorable for initial cellular adhesion and attachment [45,46]. This revealed that the cells in the CS/GelMa hydrogel adhered well to the internal channels of the scaffolds and more favorable for cellular proliferation and attachment. Similarly, Davoodi et al. modified the surface of 3D-printed titanium scaffold with cell-laden GelMa, and their results showed that cell adhesion increased with increased concentrations of GelMa [47]. Reports have been made stating that the initial levels of cellular adhesion can be used to predict subsequent cellular activities, and it has been reported that the levels of cellular adhesion at the early stages could be used as a reliable indicator for subsequent cellular proliferation and differentiation [48].

**Figure 8:**
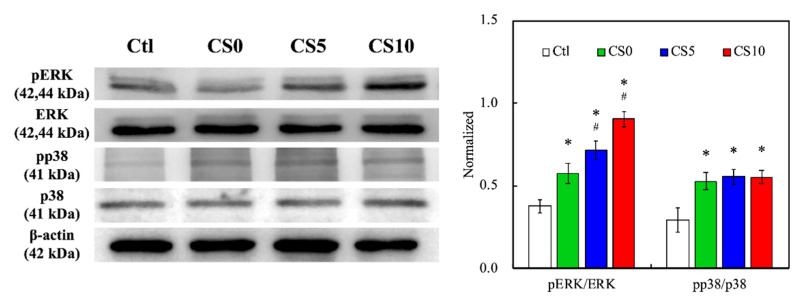
Immunodetection of anti-extracellular signal-regulated kinase 1/2 (ERK 1/2), anti-phosphoextracellular signal-regulated kinase 1/2 (pERK 1/2), anti-p38, anti-phospho p38 (pp38), and β-actin protein expression in hDPSCs-laden CS/GelMa scaffolds for 1 day. Quantification of pERK/ERK and pp38/p38. * indicates a significant difference (*p* < 0.05) when compared to Ctl. ^#^ indicates a significant difference (*p* < 0.05) when compared to CS0.

To confirm the integrin-mediated signaling pathway involved in the osteogenesis of CS scaffolds, ERK, p38, and their phosphorylation counterparts were evaluated, for which the results are shown in Figure 8.

The addition of CS into the GelMa led to enhanced activation of ERK in terms of phosphorylation after culture. CS10 and CS5 had significantly higher pERK as compared to CS0. Interestingly, a 3D model of GelMa had significantly higher pERK expression as compared to Ctl. ERK is part of a greater family known as mitogen-activated protein kinases that play a huge role as signal transducers for a wide range of cellular activities, including bone growth. Abnormalities in the ERK pathway have been found to be associated with multiple skeletal genetic abnormalities in children. In addition, studies have reported that mice with ERK1/2 gene knockout develop severe limb and head deformities. These results have prompted scientists to further investigate the functions of ERK/MAPK in bone growth, and studies have shown that the ERK/MAPK pathway is involved in in vivo and in vitro osteoblast differentiation and osteogenesis. Rodriguez-Carballo et al. showed that pERK is mainly found in osteoblasts on the bone surface, and different levels of pERK have been noted at different stages of differentiation [49]. pERK increases during the early stages of bone growth, and then peaks and gradually declines at later stages of differentiation [49]. These results suggest that ERK and pERK are involved in osteoblast differentiation and bone regeneration. Furthermore, Du et al. showed that similar molecular pathways can be found in odontogenesis processes. However, there is no significant difference of pp38 expression between the different concentrations of CS in GelMa bioinks. It was noted that 3D cell culture models exhibited increased expression of pp38 as compared to 2D cell cultures. All groups, including CS0, CS5, and CS10, had significantly higher levels of pp38 as compared to the control [50]. Greenblatt et al. showed that p38 is another crucial mediator in tooth development and enamel secretion [51]. Taken together, further studies are required to explore the effects of CS on osteo/odontogenesis since the results of the present study showed that the 3D CS/GelMa scaffolds were able to upregulate ERK and p38 phosphorylation. On the other hand, CS is a synthetic material often used for hard tissue engineering. CS is known to contain and release Ca and Si ions into its surrounding fluid. These two ions are directly involved in numerous physiological and functional bone processes. Ca is known to promote the differentiation of stem cells into osteoblasts while Si is known to enhance calcification and inhibition of resorption. Even though CS scaffolds have sufficient mechanical strength for hard tissue engineering, it being a synthetic material means it is inert as compared to GelMa. Therefore, for this study, we attempt to incorporate CS into GelMa in order to further enhance the capabilities of GelMa for hard tissue engineering. Recently, we fabricated GelMa/Si ions, and the results showed that GelMa could be further modified to enhance tissue regeneration. In that study, the presence of Si ions further enhanced p38 and ERK expression, both of which are important for downstream cellular signaling and activation.

### 3.4. Odontogenic Behaviors

**Figure 9:**
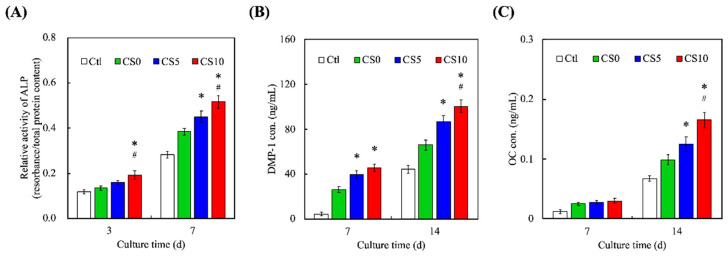
Expressions of (**A**) ALP, (**B**) DMP-1 and (**C**) OC of hDPSCs-laden CS/GelMa scaffolds for different time-points. * = significant difference as compared to CS0 and ^#^ = significant difference as compared to CS5 (*p* < 0.05). Data are presented as mean ± SEM, *n* = 6 for each group.

The effects of CS0, CS5, and CS10 scaffolds on osteogenesis and osteoblast differentiation were determined at the molecular level by investigating osteogenesis-related protein ALP, DMP-1, and OC. The results are shown in Figure 9. CS10 was found to have significantly increased secretion of ALP on both days 3 and 7 of culture, as compared to CS5 and CS0. In addition, both CS5 and CS10 were found to have significantly increased secretion of DMP-1 as compared to CS0 at all timepoints, while CS10 only had significantly increased secretion of DMP-1 on day 7, as compared to CS5. Lastly, there were no significant differences noted on day 3 for OC secretion in any of the groups, with CS10 having significant increased secretion of OC on day 7, as compared CS5 and CS0. CS-based products are highly sought after in the field of bone engineering due to its ability to release ions at a concentration that promotes osteoblast differentiation and proliferation. As mentioned earlier, Si is involved in metabolic processes involving calcification, increasing bone density, and inhibiting bone resorption. In addition to stimulating osteoblast proliferation, Si has also been reported to regulate and enhance osteogenesis-related proteins. In addition, the extracellular matrix of bone consists of mainly collagens and non-collagenous proteins such as glycoproteins and proteoglycans of which, ALP, DMP-1 and OC make up the bulk of the glycoproteins group [52]. Even though all three are related to osteogenesis, there are still some slight differences in their expressions. ALP, as its name suggests, hydrolyzes inorganic pyrophosphate, which is known to inhibit bone regeneration. In addition, ALP provides the inorganic phosphates necessary for the formation of hydroxyapatite. ALP is an early marker for osteogenesis. Its expression gradually declines as bone regeneration occurs. On the other hand, DMP-1 is mainly involved in dentin remodeling [53]. It works by binding to receptors on bone surfaces to subsequently activate osteoclasts for the purpose of remodeling. In addition, OC is a newer protein that was recently determined to also be involved in systemic functions other than bone regeneration. Regardless, OC is secreted by osteoblasts has residues that have a very high affinity for hydroxyapatite and is involved in promoting bone mineralization [54]. Among these three proteins, ALP is usually the first protein to appear in osteogenesis, followed by OPN, with OC being the last. This was also reflected in our results.

**Figure 10:**
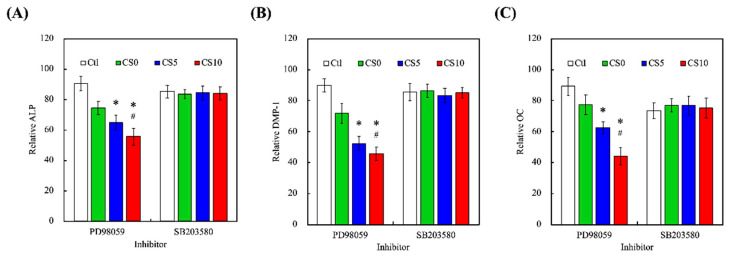
Expressions of (**A**) ALP, (**B**) DMP-1 and (**C**) OC of hDPSCs-laden CS/GelMa scaffolds after treatment of PD98059 and SB203580 inhibitors against for ERK1/2 and p38, respectively. The values of the untreated groups were used as the 100% reference level. * = significant difference as compared to CS0 and ^#^ = significant difference as compared to CS5 (*p* < 0.05). Data are presented as mean ± SEM, *n* = 6 for each group. For different time-points. * = significant difference as compared to CS0 and # = significant difference as compared to CS5 (*p* < 0.05). Data are presented as mean ± SEM, *n* = 6 for each group.

In order to confirm the role of ERK 1/2 and p38 in mediating CS-contained GelMa 3D scaffolds-induced odontogenesis, we investigated by using the specific ERK 1/2 inhibitor PD98059 and p38 inhibitor SB203580 and evaluated the odontogenic-related markers. On day 7, a significant reduction (*p* < 0.05) in ALP level of hDPCs treated with PD98059 was measured for CS10 compared with CS0 and CS5 (Figure 10A). Although SB20358 also inhibited by about 15% ALP expression, there was no significant difference between different CS-contained GelMa material. DMP-1 (Figure 10B) and OC (Figure 10C) also showed the same trend. Moreover, ALP enzyme activity, DMP-1, and OC decreased explicitly with an increasing CS amount of bioink, with an emphasis on the differences of material composition. Our present results also demonstrated the hypothesis that blocking ERK 1/2 with specific inhibitors may have an inhibitory effect on cell behaviors, further substantiating the idea that the CS-contained GelMa scaffold induced MAPK/ERK 1/2 signaling transduction pathways [46]. The results indicated the CS-contained GelMa 3D scaffolds could release Si ion stably and efficiently, then induce odontogenesis through MAPK signaling in hDPSCs and subsequently enhance the odontogenesis differentiation. Taken together, these results indeed proved that CS can enhance bone regeneration. However, this study is one of the first to further enhance the capabilities of CS-contained GelMa bioink, which is also an excellent bioscaffold for tissue engineering.

**Figure 11:**
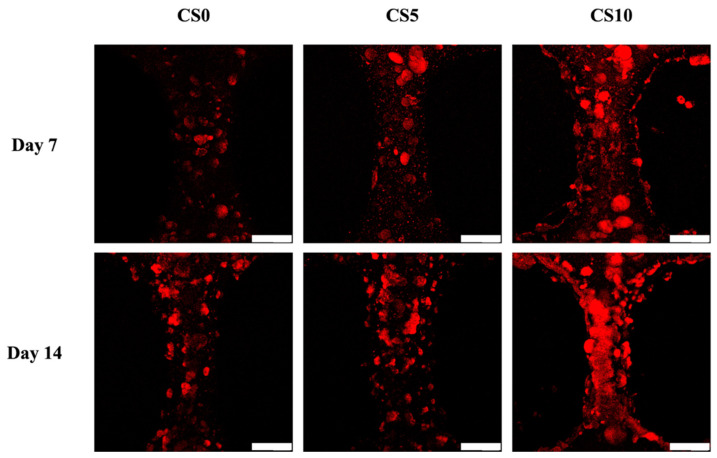
The fluorescent of Alizarin red S staining of hDPSCs-laden CS/GelMa scaffold and cultured for 7 and 14 days. The scale bar is 200 µm.

Alizarin Red S staining was performed to observe for calcium deposition for C0, C5, and C10 in Figure 10. Alizarin Red S is an anthraquinone derivative used to identify calcium-containing odontoblasts or calcium deposition. As can be seen in Figure 11, CS10 had more clusters of Alizarin Red S staining as compared to CS5 and CS0 on both day 7 and day 14, thus strongly indicating there was an increased amount of calcium mineral deposition in the CS10 group. Numerous studies have been conducted attempting to modify gelatin to improve its biocompatibility and mechanical properties in order to make it better suited for hard tissue engineering [55]. Taking the studies mentioned previously into account, it is reasonable to conclude that there is upregulation of the expression of odontogenic-related markers, subsequently mediating MAPK signaling pathways and thus improving hDPSCs odontogenesis differentiation in CS/GelMa bioscaffolds.

## 4. Conclusions

Dental injury is one of the most common issues affecting millions of people world-wide. Bioinspired stem cell-based tissue engineering has certainly gained popularity in the treatment of various clinical diseases including dental injuries. Scaffold materials and structures, as well as their properties, are important considerations in designing scaffolds. Therefore, we developed the hDPSCs-laden CS/GelMa scaffolds with various CS concentrations for the purpose of dental regeneration. As shown above, the XRD and FTIR results proved that CS was successfully incorporated into the GelMa via covalent bonds. The ability to release Si ions from the CS scaffolds contributed significantly to the enhanced regenerative effects of the scaffolds. The ability to release Si ions from the CS scaffolds contributed significantly to the enhanced regenerative effects of the scaffolds. In addition, the hDPSCs-laden CS/GelMa bioink will carry out two different signal transmission pathways for cell growth and differentiation. The first part is to use GelMa’s 3D culture environment to improve the performance of p38/MAPK and promote cell growth, and the other part is to stimulate the activation of ERK/MAPK by CS, and the concentration is positively correlated. Moreover, we also demonstrated that the release of Si ions from the CS/GelMa bioink was able to enhance the expression of various odontogenic-related biomarkers, including ALP, DMP-1, and OC. Finally, improved calcium mineralization was also observed in those groups with CS incorporation. At this point in time, it is important to note that both teeth and bone regeneration fall under hard tissue engineering since they share very similar regenerative processes. In conclusion, the incorporation of CS into GelMa bioinks not only significantly enhances the scaffold’s mechanical properties, it is also able to promote odontogenesis, as evidenced by the data discussed above. Thus, we believe that CS/GelMa scaffolds comprise a promising strategy for future dental tissue engineering as well as clinical applications.

## Data Availability

Data are available in a publicly accessible repository.
